# Monomer structure of a hyperthermophilic β-glucosidase mutant forming a dodecameric structure in the crystal form

**DOI:** 10.1107/S2053230X14010188

**Published:** 2014-06-18

**Authors:** Makoto Nakabayashi, Misumi Kataoka, Masahiro Watanabe, Kazuhiko Ishikawa

**Affiliations:** a Biomass Refinery Research Center, National Institute of Advanced Industrial Science, 3-11-32 Kagamiyama, Higashi-Hiroshima, Hiroshima 739-0046, Japan

**Keywords:** β-glucosidases, thermostable enzyme, intersubunit interactions, *Pyrococcus furiosus*

## Abstract

One of the β-glucosidases from *Pyrococcus furiosus* (BGLPf) is found to be a hyperthermophilic tetrameric enzyme that can degrade cellooligosaccharides. Recently, the crystal structures of the tetrameric and dimeric forms were solved. Here, a new monomeric form of BGLPf was constructed by removing the C-terminal region of the enzyme and its crystal structure was solved at a resolution of 2.8 Å in space group *P*1. It was discovered that the mutant enzyme forms a unique dodecameric structure consisting of two hexameric rings in the asymmetric unit of the crystal. Under biological conditions, the mutant enzyme forms a monomer. This result helps explain how BGLPf has attained its oligomeric structure and thermostability.

## Abstract 2

A protein-engineering study revealed that the C-terminal domain of a thermostable β-glucosidase contributes to its polymeric state.

## 1. Introduction   

An efficient saccharification process of cellulosic biomass by cellulases is required for the production of biofuels or bio-based materials from cellulosic biomass (Bayer & Lamed, 1992 ▶; Farrell *et al.*, 2006 ▶; Joshi & Mansfield, 2007 ▶; Ragauskas *et al.*, 2006 ▶). Cellulase systems consist of three categories of cellulase that catalyze the conversion of cellulose into glucose: endoglucanases (EGs), cellobiohydrolases (CBHs) and β-glucosidases (BGLs) (Baldrian & Valásková, 2008 ▶; Stricker *et al.*, 2008 ▶; Tomme *et al.*, 1995 ▶). BGL (BGLPf, family 1) isolated from *Pyrococcus furiosus* exhibits high hydrolytic activity towards cellooligosaccharides at high temperature (Bauer *et al.*, 1996 ▶; Kaper *et al.*, 2000 ▶). BGLPf has significant potential for the complete saccharification of cellulosic biomass at high temperature (Kim & Ishikawa, 2010 ▶). The tetrameric and dimeric BGLPf structures have been solved at resolutions of 2.35 and 1.70 Å, respectively (Kado *et al.*, 2011 ▶; Nakabayashi *et al.*, 2014 ▶). The industrial use of BGLPf will require large amounts of protein, but oligomeric enzymes are difficult to secrete in large quantities by some hosts. To address this issue, we have been trying to create a monomeric mutant that retains the intrinsic activity of the native enzyme. In a previous experiment (Nakabayashi *et al.*, 2014 ▶), we accidentally obtained the monomeric mutant BGLPf (data not published). Here, we present the biochemical and structural properties of the monomeric mutant of BGLPf and discuss its unique structure.

## 2. Materials and methods   

Unless otherwise noted, all experiments were performed at room temperature.

### 2.1. Construction of the mutant BGLPf gene   

**Figure 1:**
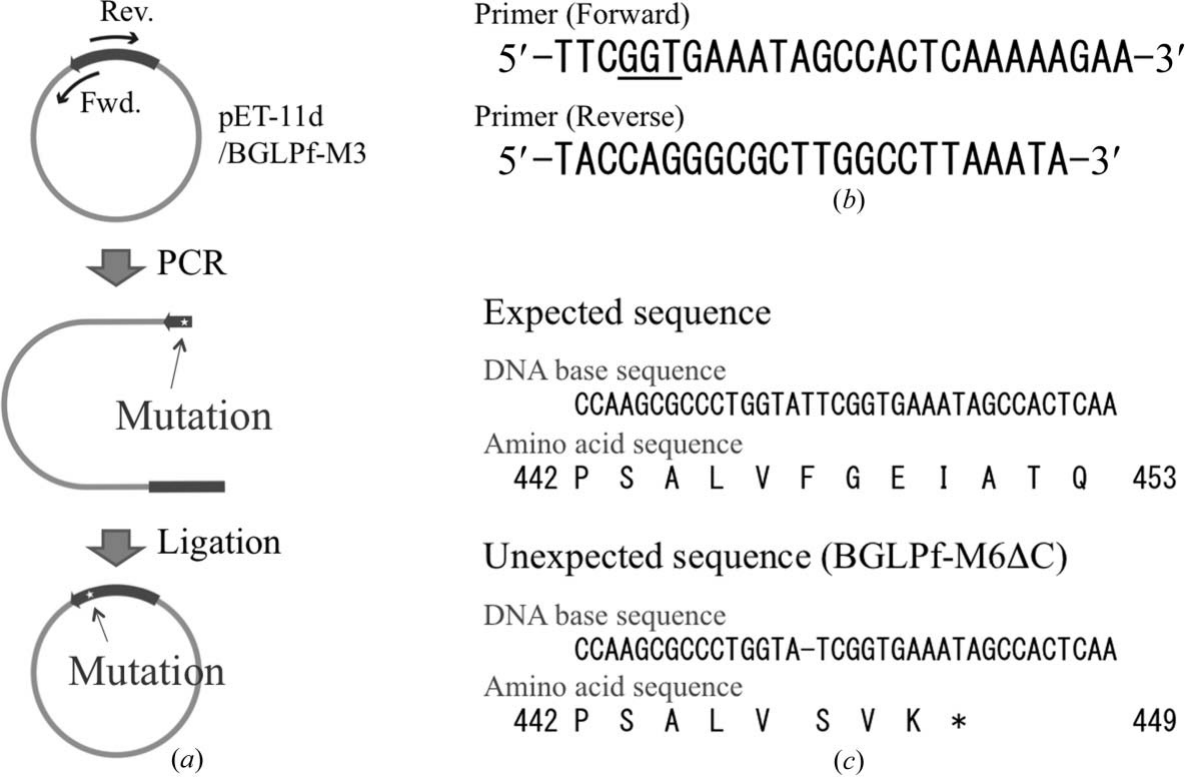
Unexpected plasmid caused by an irregular ligation. The method by which the substitutive mutations were introduced consists of two steps: (*a*) PCR with two primers whose sequences are not complementary is first performed; the self-ligation reaction of the long double-stranded DNA follows. Sequences of the primers and plasmids are shown in (*b*) and (*c*), respectively. The underlined sequence in (*b*) indicates the mutation site. Deletion of a base pair is shown in (*c*) as a hyphen. DNA and related amino-acid sequences are shown as one-letter codes. The stop codon is shown by an asterisk. Contrary to our expectation, a plasmid with a frame-shift mutation (M6ΔC) attributed to an irregular self-ligation process was found among the obtained clones.

To obtain a mutant BGLPf gene, we used the KOD -*Plus*- Mutagenesis Kit (Toyobo) and performed PCR with the pET11d/BGLPf-M3 plasmid (Nakabayashi *et al.*, 2014 ▶) as a template followed by a self-ligation step. BGLPf-M3 is a mutant in which Arg170, Arg220 and Tyr227 are substituted with Ala, Ala and Phe, respectively. Two primers, 5′-TTC**GGT**GAAATAGCCACTCAAAAAGAA-3′ (mutation site in bold) and 5′-TACCAGGGCGCTTGGCCTTAAATA-3′, were employed for PCR to create an additional substitutive mutation of R448G in BGLPf-M3 (Fig. 1 ▶). Contrary to our expectation, a plasmid with a frame-shift mutant attributed to an irregular self-ligation process was accidentally obtained from one of the clones (Fig. 1 ▶). The plasmid obtained is translated to create a mutant BGLPf, BGLPf-M6ΔC, that lacks the C-terminal 23 residues and includes six substitutive mutations R170A, R220A, Y227F, F447S, R448V and E449K. We took the opportunity to use this plasmid to express the C-terminal deletion mutant BGLPf.

### 2.2. Protein expression and purification   

The recombinant mutants were expressed in *Escherichia coli* BL21 (DE3) cells (Novagen). Cell cultures were grown at 37°C in Luria Broth medium containing 100 mg ml^−1^ ampicillin sodium salt until the optical density at 600 nm (OD_600_) reached 0.3. Cell cultures subsequently were grown at 16°C until the OD_600_ reached 0.6 and were induced for 6 h with 1 m*M* isopropyl β-d-1-thiogalactopyranoside (IPTG) at 16°C.

The harvested cells for BGLPf-M6ΔC were lysed on ice by sonication in 50 m*M* Tris–HCl pH 8.0 with 2 m*M* phenylmethylsulfonyl fluoride. The cell lysate was centrifuged at 9000*g* for 30 min at 4°C. The supernatant was fractionated with ammonium sulfate up to 80% saturation. After centrifugation, the pellet was resuspended in 50 m*M* Tris–HCl pH 8.0 and then dialyzed overnight at 4°C against 50 m*M* Tris–HCl pH 8.0. The filtered lysate was loaded onto a HiTrap Q anion-exchange column (GE Healthcare Biosciences) equilibrated with 50 m*M* Tris–HCl pH 8.0 and eluted with a linear gradient of 0–1.0 *M* NaCl. The solution eluted was fractionated with ammonium sulfate up to 35% saturation. After centrifugation, the pellet was resuspended in 20 m*M* Tris–HCl pH 8.0 containing 0.1 *M* NaCl. The lysate was filtered and loaded onto a HiLoad 26/60 Superdex 200 pg (GE Healthcare Biosciences) with 20 m*M* Tris–HCl buffer pH 8.0 containing 0.1 *M* NaCl.

The other recombinant, BGLPf-M4b (Nakabayashi *et al.*, 2014 ▶), was purified using a similar method as for BGLPf-M6ΔC except that following the HiTrap Q chromatography the supernatant of ammonium sulfate fractionation up to 40% saturation was loaded onto the Hi-Load 26/60 Superdex 200 pg.

The purity and size of the proteins were assessed by reducing SDS–PAGE. The concentrations of the BGLPfs were determined from UV absorbance at 280 nm using molar extinction coefficients as calculated from their protein sequences using a standard method (Gill & von Hippel, 1989 ▶).

### 2.3. Crystallization   

**Figure 2:**
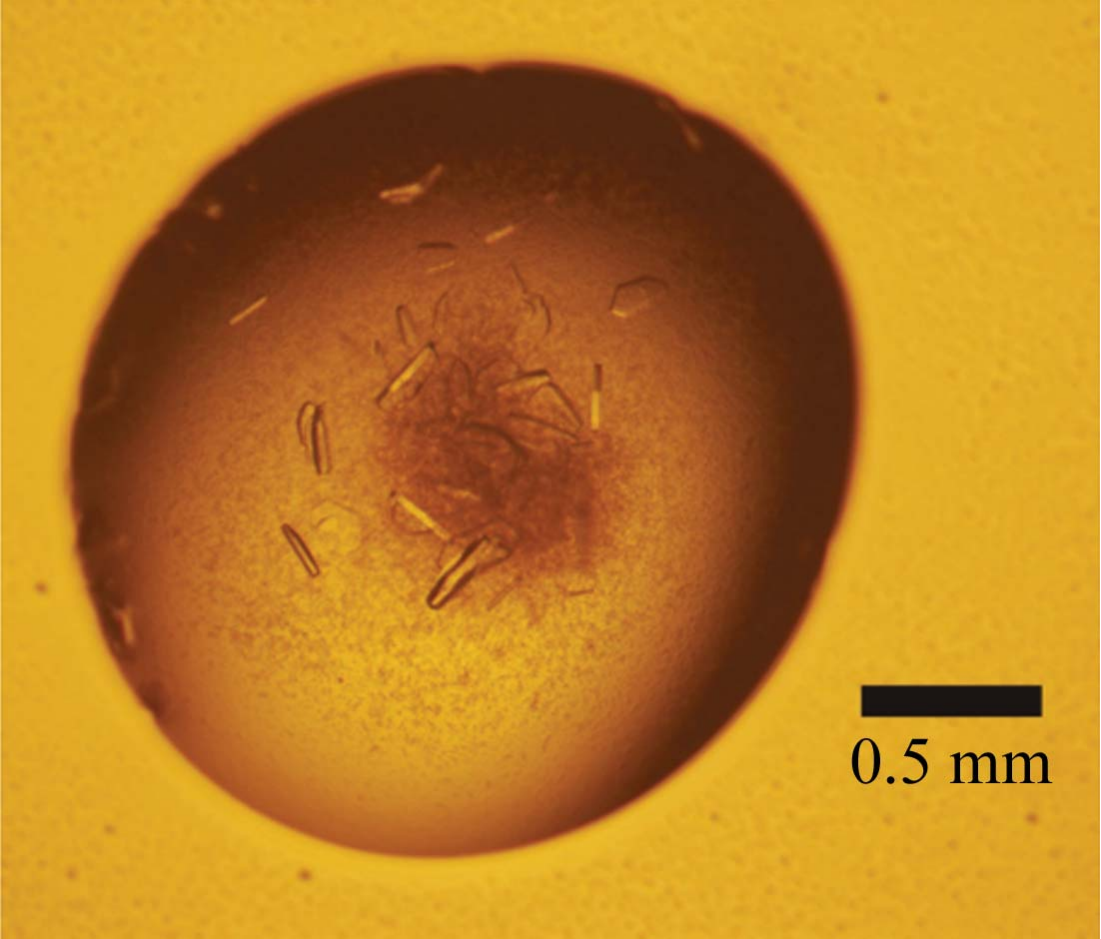
Crystals of BGLPf-M6ΔC. Precipitant solution consisting of 0.1 *M* HEPES–NaOH pH 7.0, 0.2 *M* calcium acetate, 20% PEG 3350, 5% ethylene glycol was used for BGLPf-M6ΔC. Crystallization was performed by the hanging-drop vapour-diffusion method.

Purified BGLPf-M6ΔC solution was concentrated to about 7 mg ml^−1^ by ultrafiltration. The solution was allowed to crystallize by the hanging-drop vapour-diffusion method using a series of precipitant solutions consisting of HEPES–NaOH pH 7.0–7.8, calcium acetate, PEG 3350, ethylene glycol. Droplets for crystallization were prepared by mixing 2.3 µl protein solution and 1.0 µl precipitant solution, and the droplets were equilibrated against 500 µl precipitant solution at 20°C. It took about one week to obtain crystals with X-ray diffraction quality (Fig. 2 ▶). The crystal that was used in the diffraction experiment was obtained using a precipitant solution consisting of 0.1 *M* HEPES–NaOH pH 7.6, 0.2 *M* calcium acetate, 20% PEG 3350, 5% ethylene glycol.

### 2.4. Diffraction experiments and structure analysis   

Prior to diffraction data collection, crystals of M6ΔC were soaked in cryoprotectant solutions which were made based on the precipitants and contained 10 or 20%(*v*/*v*) ethylene glycol. The crystals were first soaked in the solution with 10% ethylene glycol and were then soaked in the solution with 20% ethylene glycol. Diffraction data sets were collected at −173°C in a stream of nitrogen gas on beamline BL44XU of SPring-8, Hyogo, Japan. Reflections were recorded with an oscillation range per image of 1°. Diffraction data were indexed, integrated and scaled using *HKL*-2000 (Otwinowski & Minor, 1997 ▶).

The structure of BGLPf-M6ΔC was solved by molecular replacement with *Phaser* (McCoy *et al.*, 2007 ▶). One of four chains in the coordinates of dimeric BGLPf (PDB entry 3wdp chain *P*; Nakabayashi *et al.*, 2014 ▶) was isolated and used as a search model for molecular replacement. Finalized sets of atomic coordinates were obtained after iterative rounds of model modification with *Coot* (Emsley *et al.*, 2010 ▶) and refinement with *REFMAC*5 (Murshudov *et al.*, 2011 ▶) and *CNS* (Brünger *et al.*, 1998 ▶) by rigid-body refinement, positional minimization, water molecule identification and individual isotropic *B*-value refinement. 126 water molecules and no ligand molecules were inserted in the coordinates. Superimposition between the structure models was carried out using the program *ProFit* (http://www.bioinf.org.uk/software/profit). Pictures of the BGLPfs were drawn using the program *PyMol* (http://www.pymol.org).

### 2.5. Evaluation of molecular sizes   

The oligomeric state of the mutants and wild-type BGLPf (BGLPf-WT) were examined by gel filtration using a HiLoad 26/60 Superdex 200 pg column and the dynamic light-scattering (DLS) method (instrument custom-built by Associate Professor Shinpei Tanaka, Hiroshima University, Hiroshima, Japan).

The samples of the mutants and the wild type were loaded in the gel filtration with 50 m*M* Tris–HCl buffer pH 8.0 containing 0.15 *M* NaCl. The flow rate was adjusted to 2.0 ml min^−1^ and the time course of the absorbance at 280 nm was monitored. The samples of the mutants and the wild type were adjusted at a concentration of 10 mg ml^−1^ with 20 m*M* Tris–HCl buffer pH 8.0 for the DLS. The DLS measurements were performed at 20°C.

### 2.6. Evaluation of thermostabilities   

Differential scanning calorimetry (DSC) measurements were carried out using a nanoDSCII instrument (TA Instruments, Delaware, USA) with platinum tubing cells with a volume of 0.3 ml by Associate Professor Harumi Fukada, Osaka Prefecture University, Osaka, Japan. Prior to the DSC experiment, the samples of the mutants and the wild type were dialyzed against 50 m*M* sodium phosphate buffer pH 7.0 and were adjusted to a concentration of 10 mg ml^−1^. The experiments were performed over a temperature range of 35–125°C at a scan rate of 1°C min^−1^.

Prior to measurement of residual activities of the mutants and the wild type, the purified enzymes were incubated for 10 min in 50 m*M* Tris–HCl buffer pH 7.2 at the given temperatures (50, 60, 65, 70, 75, 80, 85 and 90°C). The residual activity of the mutant or the wild type after heating was assayed under a standard condition containing the enzyme at 0.075 mg ml^−1^ and 10 m*M* cellobiose for 10 min at 40°C. The activity was expressed as the concentration of glucose produced as a percentage (Kim & Ishikawa, 2010 ▶).

## 3. Results and discussion   

### 3.1. Preparing a mutant from an unexpected frame-shift mutation   

The hyperthermophilic β-glucosidase from *P. fusiosus* (BGLPf) was discovered by Bauer *et al.* (1996 ▶) and its structural model was constructed by Kado *et al.* (2011 ▶). The structure of BGLPf shows a stable homotetrameric structure forming 222 point-group symmetry. Mutagenesis to disrupt the tetrameric structure was performed, and a mutant enzyme with three substitutive mutations (BGLPf-M3) was thus created and its structure was solved (Nakabayashi *et al.*, 2014 ▶). Gel-filtration analysis and DLS clarified that BGLPf-M3 forms a dimeric structure in aqueous solution. In contrast, BGLPf-M3 forms two distinct types of pseudo-tetrameric structures that consist of two types of dimers, *PQ* and *RS*, in the crystal (Nakabayashi *et al.*, 2014 ▶; see Supplementary Fig. S1^1^). In order to obtain a monomeric BGLPf, it should be noted that the C-terminal residues tether the dimers *PQ* and *RS*, because superimposing the main chains slightly discriminates the C-terminus from Pro457 to Lys472 in monomers *P*, *Q*, *R* and *S* (Supplementary Fig. S2). Thus, the C-terminal region seems to be important for stable dimer formation according to previously performed structural analysis of BGLPf-M3 (Nakabayashi *et al.*, 2014 ▶).

In our many protein-engineering experiments with BGLPf, we accidentally obtained a mutant gene (BGLPf-M6ΔC) without the C-terminal region from the BGLPf-M3 gene. Our method for site-directed mutation, which employs the KOD -*Plus*- Mutagenesis Kit (Toyobo), includes a ligation reaction between blunt ends of long double-stranded DNA, and an anomalous ligation with one missing base pair accidentally occurred. This mutation gives rise to a complicated result, a frame-shift mutation with three substitutions and a subsequent nonsense mutation (Fig. 1 ▶). We found that BGLPf-M6ΔC lacks the C-terminal 23 residues of BGLPf-M3 and includes an additional three substitutions (F447S, R448V and E449K; Fig. 1 ▶). The mutant BGLPf-M6ΔC was expressed in *E. coli* and purified by a standard method.

### 3.2. Crystal structure of BGLPf-M6ΔC   

**Figure 3:**
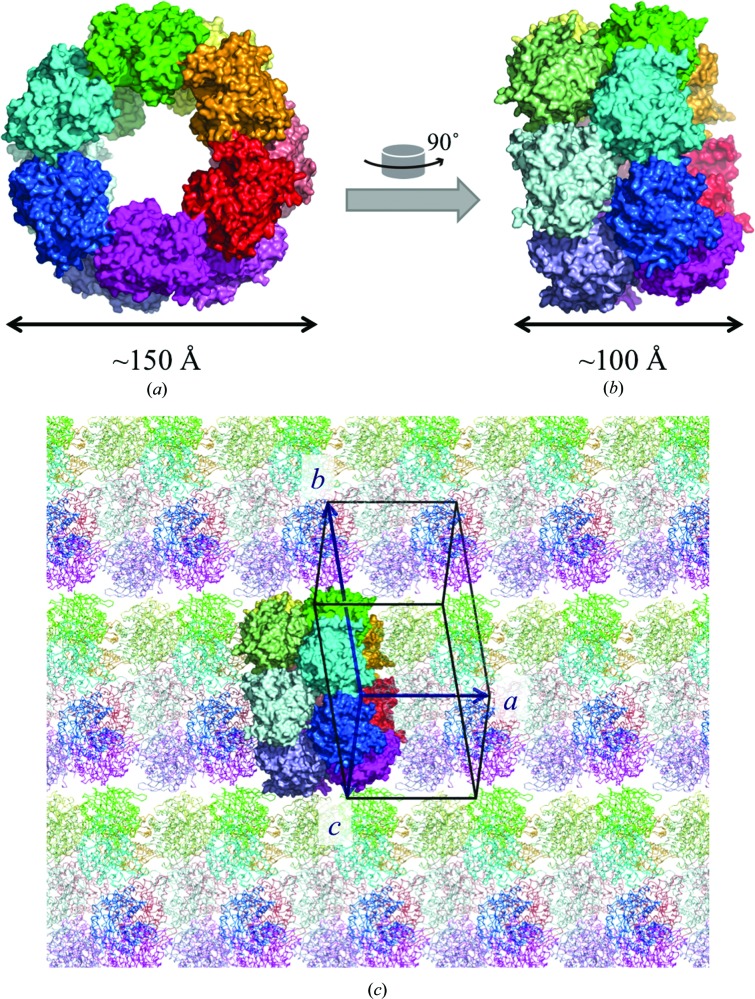
Crystal structure of BGLPf-M6ΔC in space group *P*1. The packing of molecules in the asymmetric unit resembles duplicated hexameric rings (*a*), (*b*) in which a noncrystallographic sixfold axis is embedded. 12 subunits (*A*, red; *B*, pale red; *C*, bright orange; *D*, pale yellow; *E*, green; *F*, pale green; *G*, cyan; *H*, pale cyan; *I*, blue; *J*, light blue; *K*, magenta; *L*, violet) are shown as space-filling models in the asymmetric unit. (*a*) was rotated 90° around the vertical axis to generate (*b*). (*c*) shows the molecules on the *xy* plane of the orthogonal coordinates. One set of the 12 subunits is shown as space-filling models in the asymmetric unit. The other molecules related by crystallographic symmetry are shown as chain-trace models. A unit cell and its three lattice vectors are shown by black solid lines and dark blue arrows, respectively. The arrangement shows the reason why the space group is *P*1. The local noncrystallographic sixfold axis is not parallel to the translation vector *a*.

BGLPf-M6ΔC was crystallized using the hanging-drop vapour-diffusion method in precipitant solution containing calcium acetate, PEG 3350 and ethylene glycol, which are different conditions than those used for BGLPf-WT and BGLPf-M3 as reported previously. Structural analysis showed that the BGLPf-M6ΔC crystal belonged to the space group *P*1, which is different from those of BGLPf-WT and BGLPf-M3. Diffraction data were collected and refined to a resolution of 2.81 Å (Table 1 ▶). 12 molecules (chain IDs *A*, *B*, *C*, *D*, *E*, *F*, *G*, *H*, *I*, *J*, *K* and *L*) per asymmetric unit gave a crystal volume per protein mass (Matthews, 1968 ▶; *V*_M_) of 2.91 Å^3^ Da^−1^ and a solvent content of 57.7%(*v*/*v*), which is lower than that for BGLPf-WT and BGLPf-M3. The asymmetric unit exhibits a unique duplicated hexameric ring structure that includes the 12 monomers; one of the rings includes six monomers named *A*, *C*, *E*, *G*, *I* and *K*, and the other also includes six monomers named *B*, *D*, *F*, *H*, *J* and *L*. The arrangement of monomers therefore generates a local sixfold symmetrical axis that is not parallel to any of the crystal axes *a*, *b* and *c*, and hence the space group is *P*1 (Fig. 3 ▶). Analysis of the protein interfaces by *PISA* did not reveal sufficient interactions that could result in the formation of stable quaternary structures. Thus, the structures of monomers *A*, *B*, *C*, *D*, *E*, *F*, *G*, *H*, *I*, *J*, *K* and *L* are able to form a pseudo-dodecameric structure in the crystal, a complex that is presumably difficult to form in aqueous solution. Many atoms of subunits *F* and *J* in the 12 monomers were assigned especially high temperature factors. There are interactions that contribute to crystal packing, and several important interactions have poor contact areas of less than 300 Å^2^ as indicated by *PISA* (see Supplementary Table S1). The effect of crystal packing on diffraction data quality and intermolecular interactions should be significant (Mizutani *et al.*, 2008 ▶). Since several BGLPf-M6ΔC interactions involved in the packing have only poor contact areas, the crystal was less stable than those of BGLPf-WT and BGLPf-M3. Using the low-resolution diffraction data collected up 2.8 Å resolution, the monomer structure was refined (Table 1 ▶). We attribute the low-resolution data to poorly ordered lattice molecules due to unstable intermolecular interactions in the crystal.

The deletion of the C-terminal region of BGLPf, which drastically altered the crystal packing into space group *P*1, did not however affect the individual structures of monomeric BGLPf. The root-mean-square (r.m.s.) deviations of the C^α^-atom positions (2–449) of subunits *B*, *C*, *D*, *E*, *F*, *G*, *H*, *I*, *J*, *K* and *L* compared with subunit *A* in the asymmetric unit were less than 0.4 Å. Furthermore, the r.m.s. deviations of the C^α^-atom positions (2–449) of subunits *A*, *B*, *C*, *D*, *E*, *F*, *G*, *H*, *I*, *J*, *K* and *L* of BGLPf-M6ΔC compared with subunit *A* of BGLPf-WT were between 0.44 and 0.71 Å, even though the region Asp96–Val108 showed a small mismatch of more than 2 Å. These results indicate that the overall structure of the monomer is not influenced by the deletion and suggest that BGLPf-M6ΔC can reliably be prepared as a monomer.

### 3.3. Dimerization contributes to thermostability   

The oligomeric state of BGLPf-M6ΔC was determined using gel-filtration analysis. Ferritin (440 kDa), aldolase (158 kDa), conalbumin (75 kDa) and ovalbumin (44 kDa) were used as standard markers. The results indicated that BGLPf-M6ΔC (63 kDa) is monomeric in aqueous solution. The dimeric state of BGLPf-M3 and the tetrameric state of BGLPf-WT have also been determined (Supplementary Fig. S3). We next employed DLS to evaluate the sizes of the mutants. The hydrodynamic radius of BGLPf-M6ΔC was estimated to be 3.1 nm (Supplementary Fig. S4). This result is consistent with that obtained from gel-filtration chromatography.

**Figure 4:**
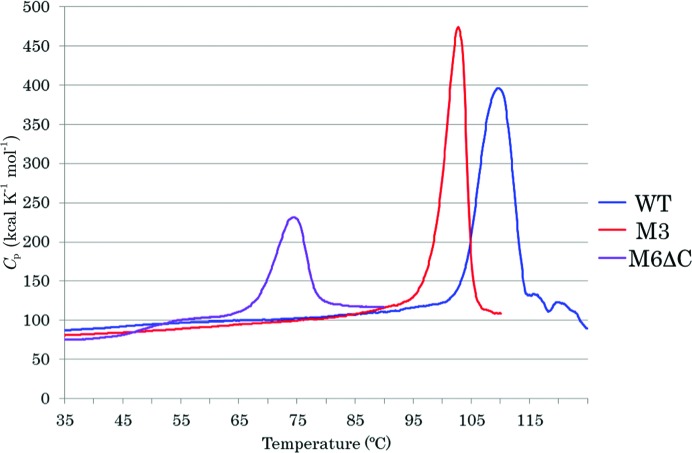
Thermal stability of BGLPf-WT (WT), BGLPf-M3 (M3) and BGLPf-M6ΔC (M6ΔC). DSC results are shown as excess heat capacity, *C*_p_ (kcal K^−1^ mol^−1^), *versus* temperature (°C) profiles. Proteins (1.0 mg ml^−1^) were dissolved in 50 m*M* sodium phosphate buffer pH 7.0. The *T*_m_ values of BGLPf-WT, BGLPf-M3 and BGLPf-M6DC were estimated as 109.5, 102.0 and 74.5°C, respectively. The data for WT and M3 are from Nakabayashi *et al.* (2014 ▶).

**Figure 5:**
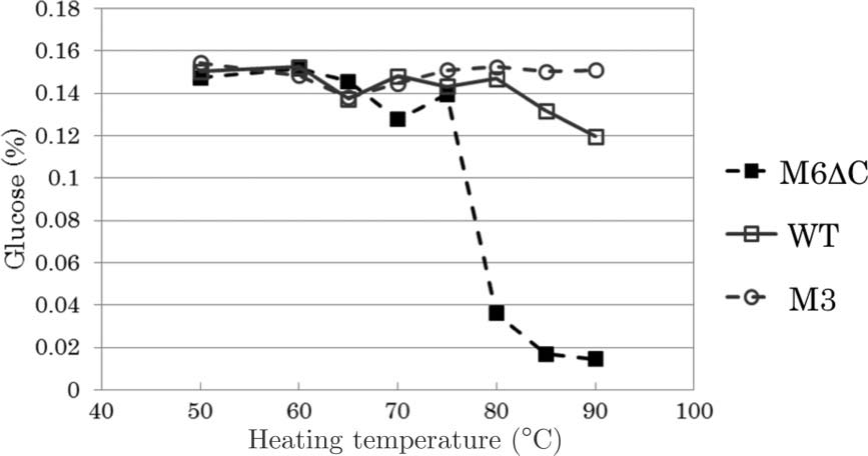
Residual activities of BGLPf-M6ΔC (M6ΔC), BGLPf-WT (WT) and BGLPf-M3 (M3) after heating. Purified enzymes (1.0 mg ml^−1^) were incubated for 10 min in 50 m*M* Tris–HCl buffer pH 7.2 at the given temperatures. The residual activity of each enzyme after heating was assayed under standard conditions with 10 m*M* cellobiose for 10 min at 40°C. Activity is expressed as the concentration of glucose produced (%). The data for WT and M3 are from Nakabayashi *et al.* (2014 ▶).

DSC was used to examine the thermostability of the mutants (1.0 mg ml^−1^ in 50 m*M* sodium phosphate buffer pH 7.0). The monomeric mutant BGLPf-M6ΔC had a melting temperature (*T*_m_) of 74.5°C (Fig. 4 ▶). Tetrameric BGLPf-WT and dimeric BGLPf-M3 had *T*_m_ values of 110 and 102°C, respectively. We also evaluated the residual activity after heating to confirm the thermostability of the substitutive mutant. Enzyme solution (1.0 mg ml^−1^ in 50 m*M* Tris–HCl buffer pH 7.2) was kept at various temperatures for 10 min, and the activities were assayed. BGLPf-M6ΔC was immediately inactivated between 75 and 80°C, as shown in Fig. 5 ▶. BGLPf-WT and BGLPf-M3 were stable beyond 85°C. These data are consistent with the *T*_m_ values measured by DSC.

A previous study of BGLPf-M3 suggested that several substitutive mutations could convert the dimeric BGLPf into a monomer, and we verified this by introducing the substitutions R448E, E449R or E459G. In addition, the present study shows that conversion from a dimer to a monomer can be achieved not only by substitutions but also by a deletion. A study concerning mesophilic BGLs from the bacterium *Clostridium cellulovorans*, the fungus *Trichoderma reesei* and the termite *Neotermes koshunensis* (Jeng *et al.*, 2011 ▶) also demonstrates this principle: all of these enzymes possess shortened C-termini and are monomers. BGLPf-M4a, BGLPf-M4b and BGLPf-M4c (Nakabayashi *et al.*, 2014 ▶) obtained previously do not exhibit dimeric states, BGLPf-M4a and BGLPf-M4c are monomeric and MBGLPf-4b exists in an equilibrium between monomeric and dimeric states. All of these, which have C-terminal regions, have lower *T*_m_ values than the dimeric BGLPf-M3. The monomeric BGLPf-M6ΔC with a shortened C-terminus has a *T*_m_ value of 74.5°C, which is comparable to those of BGLPf-M4a, BGLPf-M4b and BGLPf-M4c with long C-termini. Therefore, the results obtained so far demonstrate that thermostability is not determined by the C-terminal length of BGL, but rather depends on its polymeric state. Thus, we presume that polymerization of BGL protects it against high temperatures.

## 4. Conclusion   

The hyperthermophilic β-glucosidase from *P. furiosus* forms a stable tetrameric structure. We successfully constructed a monomeric form of the enzyme by removing its C-terminal region and solved the crystal structure. This study shows that the lack of the C-terminal region does not affect the activity of the enzyme, but disrupts its oligomeric state and hyperthermostability. Furthermore, we found that the mutant enzyme can form a unique dodecameric structure consisting of two hexameric rings in its crystal form.

## Supplementary Material

Supporting Information.. DOI: 10.1107/S2053230X14010188/gx5225sup1.pdf

PDB reference: BGLPf, 3wq8

## Figures and Tables

**Table 1:** Summary of statistics and refinement of BGLPf-M6ΔC crystallographic data Values in parentheses are for the highest resolution shell.

PDB code	3wq8
X-ray source	BL44XU, SPring-8
Wavelength (Å)	0.90000
Space group	*P*1
Unit-cell parameters (Å, °)	*a* = 97.37, *b* = 148.87, *c* = 148.56, α = 120.08, β = 94.00 γ = 99.70
Resolution range (Å)	50.00–2.81 (2.90–2.81)
Total No. of reflections	592539
No. of unique reflections	170856
Completeness (%)	96.8 (92.5)
Mean *I*/σ(*I*)	28.7 (3.3)
*R*_merge_ †	0.110 (0.471)
*R*_p.i.m._ ‡	0.066 (0.301)
Refinement statistics	
No. of atoms	
Protein	43636
Water	126
Resolution range (Å)	50.00–2.81 (2.90–2.81)
*R* factor (*R*_free_/*R*_work_)§	0.275/0.243 (0.389/0.342)
Wilson *B* factor (Å^2^)	49.0
R.m.s.d., bond distance (Å)	0.013
R.m.s.d., bond angle (°)	1.480
Mean overall *B* factor (Å^2^)	61.0
Ramachandran plot (%)	
Preferred	94.18
Allowed	5.07
Outliers	0.75

† *R*_merge_ = 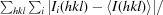 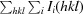, where 〈*I*(*hkl*)〉 is the mean intensity of all reflections equivalent to reflection *hkl*.

‡ *R*_p.i.m._ (Weiss, 2001 ▶) = 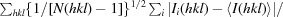 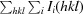, where *N*(*hkl*) is the mean redundancy.

§ *R*_work_ (*R*_free_) = 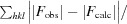 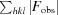, where 5% of randomly selected data were used for *R*_free_.

## Footnotes

1 Supporting information has been deposited in the IUCr electronic archive (Reference: GX5225).
