# Supplementary material for: Monomer structure of a hyperthermophilic β-glucosidase mutant forming a dodecameric structure in the crystal form
Source: Acta Crystallogr F Struct Biol Commun. 2014 Jun 18;70(Pt 7):854–9. doi: 10.1107/S2053230X14010188 (PMC4089520; doi:10.1107/S2053230X14010188)
Supplement: Supplementary file 1 [file f-70-00854-sup1.pdf]

Acta Crystallographica Section F

Volume 70 (2014)

Supporting information for article:

Monomer structure of hyperthermophilic β-glucosidase mutant forming a dodecameric structure in the crystal form

Makoto Nakabayashi, Misumi Kataoka, Masahiro Watanabe and Kazuhiko Ishikawa

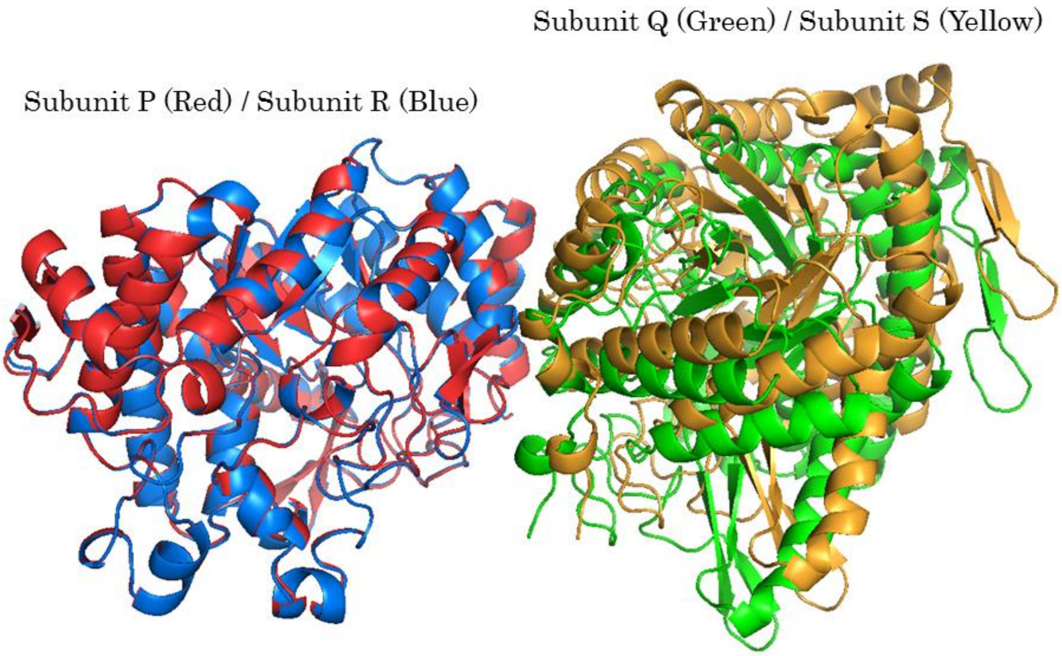
Subunit Q (Green) / Subunit S (Yellow)
Subunit P (Red) / Subunit R (Blue)

(A)

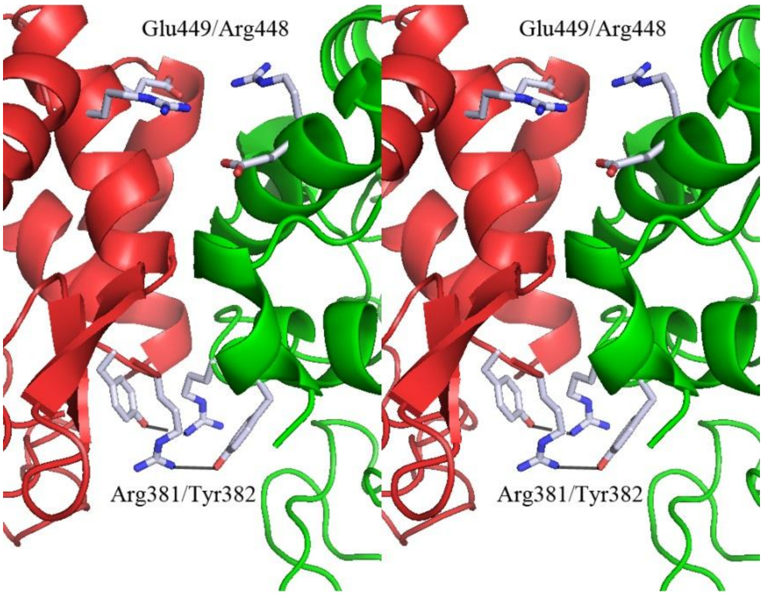
Glu449/Arg448
Arg381/Tyr382
Glu449/Arg448
Arg381/Tyr382

(B)

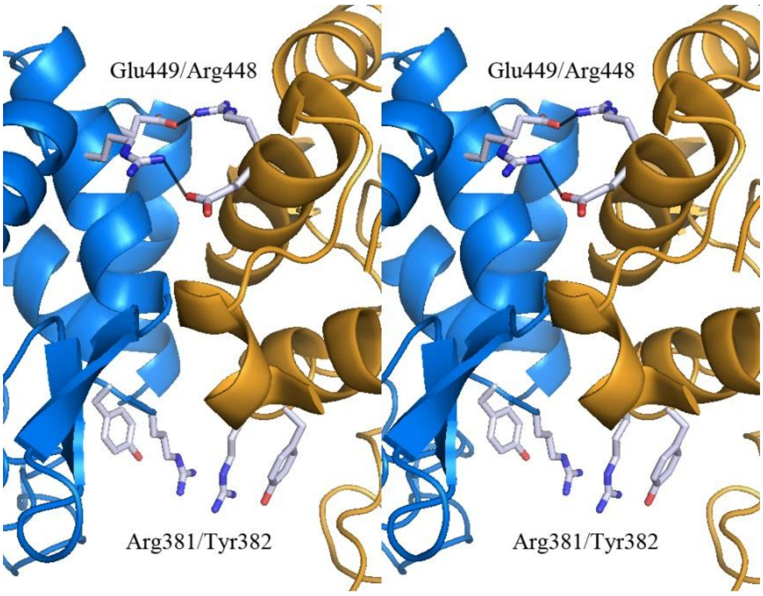

(C)

Figure S1 Comparing the RS and PQ dimer in BGLPf-M3. Subunits P, Q, R and S are shown in red, green, blue and yellow, respectively (S1A, S1B and S1C). There is a difference between the PQ- and the RS-dimer, even though the monomeric structures of P, Q, R and S are almost identical. When subunit R was superimposed on P, parts of S could not fit onto Q (S1A). Arg381 and Tyr382 interact, whereas Arg448 and Glu449 displace each other in dimer PQ (S1B, stereo diagram). Similarly, Arg448 and Glu449 interacted whereas Arg381 and Tyr382 displaced each other in dimer RS (S1C, stereo diagram). Putative interactions are shown by black solid lines. These figures and figure captions were extracted from the figure 4 in our previous article, Nakabayashi et al. *Acta Cryst.* (2014) **D70**, 877-888.

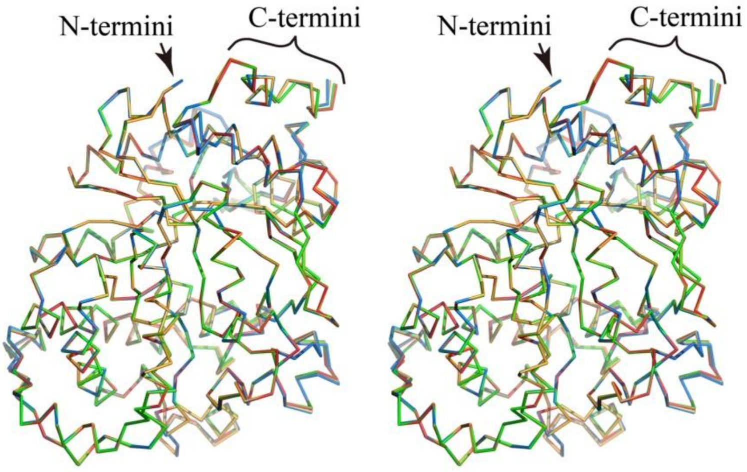

Figure S2 Superimposed Cα atoms of the four subunits, P, Q, R, and S, in BGLPf-M3 (stereo diagram). Crystals of BGLPf-M3 include two types of pseudo-tetramers; one is PP'QQ' and the other is RR'SS'. The N- and C-termini are indicated by the arrow and the curly bracket, respectively. Interestingly, superimposition between the four subunits, P (red), Q (green), R (blue), and S (yellow), shows the C-termini somewhat mismatched. The C-termini of R and S are discriminated from the others, suggesting that the region helps maintain the oligomeric structure.

Table S1 List of contact interfaces contributing to crystal-packing in BGLPf-M6ΔC

The surface area of each subunit and the interface area between two specific subunits are shown in Å^2. $\Delta^i G$ indicates the solvation free energy gain upon formation of the interface, shown in kcal/mol. The $\Delta^i G$ value is calculated as the difference in total solvation energy of the isolated and interfacing structures. An interaction with a negative $\Delta^i G$ value indicates that the interaction requires energy to dissociate. Several interactions highlighted by yellow background, in which the symmetry operations are indicated as 'x-1, y, z' and 'x-1, y-1, z-1,' contribute to crystal-growth along the translation vector *a*. All of the seven interactions, M6ΔC-19, 28, 29, 30, 32, 33 and 34, show small interface areas less than 300 Å^2, which suggest that the crystal-packing along the vector *a* in BGLPf-M6ΔC is rather weak. These data were generated by the program PISA (Krissinel & Henrick, 2007).

	Structure 1		Structure 2			Interface Area (Å^2)	$\Delta^i G$ (kcal/mol)
Interaction ID	Subunit	Surface (Å^2)	Subunit	Symmetry Operation	Surface (Å^2)		
M6ΔC-1	D	17498	C	x,y,z	17662	660.7	-10.2
M6ΔC-2	B	17696	A	x,y,z	17830	635.1	-10.7
M6ΔC-3	F	17630	E	x,y,z	17809	610.2	-8.8
M6ΔC-4	L	17986	K	x,y,z	17562	601.2	-8.8
M6ΔC-5	H	17934	G	x,y,z	18025	658.1	-11.6
M6ΔC-6	J	17754	I	x,y,z	17651	522.3	-9.2
M6ΔC-7	C	17662	I	x,y,z-1	17651	620.5	1.4
M6ΔC-8	A	17830	G	x,y-1,z-1	18025	618.7	3.2
M6ΔC-9	K	17562	E	x,y-1,z	17809	617.9	1.0
M6ΔC-10	K	17562	A	x,y,z	17830	481.8	-2.3

M6ΔC-11	I	17651	G	x,y,z	18025	479.9	-2.5
M6ΔC-12	E	17809	C	x,y,z	17662	466.5	-2.8
M6ΔC-13	G	18025	E	x,y,z	17809	463.8	-1.5
M6ΔC-14	C	17662	A	x,y,z	17830	439.2	-3.3
M6ΔC-15	K	17562	I	x,y,z	17651	419.7	-2.8
M6ΔC-16	F	17630	D	x,y,z	17498	433.9	-3.6
M6ΔC-17	L	17986	B	x,y,z	17696	433.6	-2.3
M6ΔC-18	J	17754	H	x,y,z	17934	430.3	-3.2
M6ΔC-19	D	17498	C	x-1,y,z	17662	293.2	-0.1
M6ΔC-20	L	17986	D	x,y-1,z	17498	279.2	0.1
M6ΔC-21	D	17498	H	x,y,z-1	17934	272.8	1.1
M6ΔC-22	L	17986	H	x,y-1,z-1	17934	136.5	1.6
M6ΔC-23	D	17498	B	x,y,z	17696	245.7	-0.4
M6ΔC-24	H	17934	F	x,y,z	17630	195.6	-0.6
M6ΔC-25	E	17809	D	x,y,z	17498	215.7	-1.2
M6ΔC-26	I	17651	H	x,y,z	17934	164.8	-1.7
M6ΔC-27	L	17986	A	x,y,z	17830	145.4	-1.3
M6ΔC-28	L	17986	K	x-1,y,z	17562	172.8	0.0
M6ΔC-29	H	17934	G	x-1,y,z	18025	171.4	-0.2
M6ΔC-30	H	17934	I	x-1,y,z	17651	150.5	0.3
M6ΔC-31	C	17662	B	x,y,z	17696	133.1	-0.3
M6ΔC-32	L	17986	G	x-1,y-1,z-1	18025	129.8	1.2
M6ΔC-33	J	17754	I	x-1,y,z	17651	128.9	-0.3

M6ΔC-34	J	17754	K	x-1,y,z	17562	121.2	-1.3
M6ΔC-35	J	17754	F	x,y-1,z	17630	114.1	0.4
M6ΔC-36	L	17986	J	x,y,z	17754	109.9	0.6

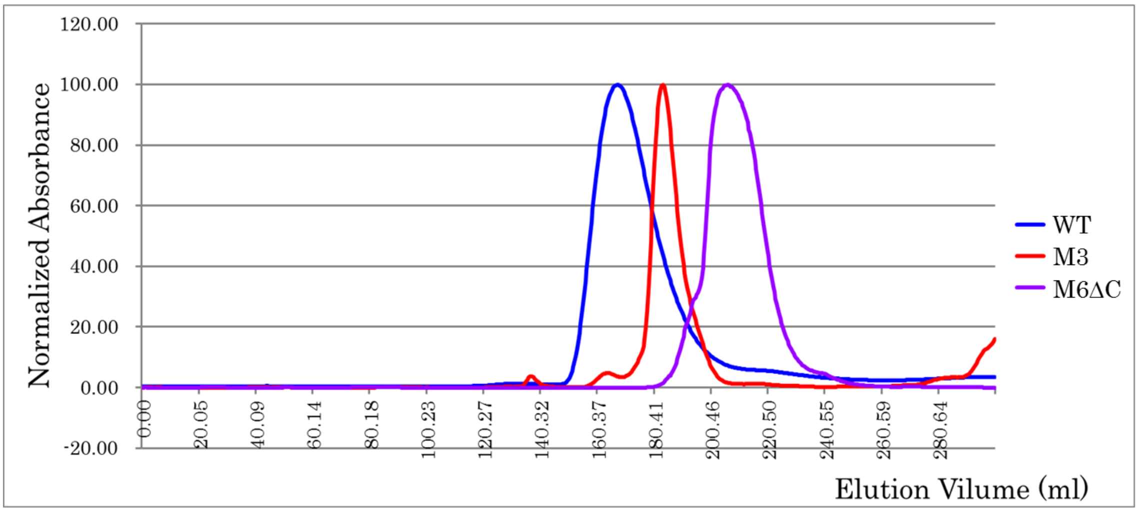

Figure S3 Gel-filtration profiles of the BGLPf-WT, M3 and M6ΔC. Elution profiles of the BGLPf-WT, M3 and M6ΔC are shown by blue, red and purple lines, respectively. Elution volumes of the WT, M3 and M6ΔC obtained from the profiles are 167.62, 183.40 and 206.43 ml, respectively. Molecular masses of the WT, M3 and M6ΔC estimated from the elution volumes are 236 kDa, 136 kDa and 61 kDa, respectively. The data for the WT and M3 are referred from our previous article, Nakabayashi et al. *Acta Cryst.* (2014) **D70**, 877-888.

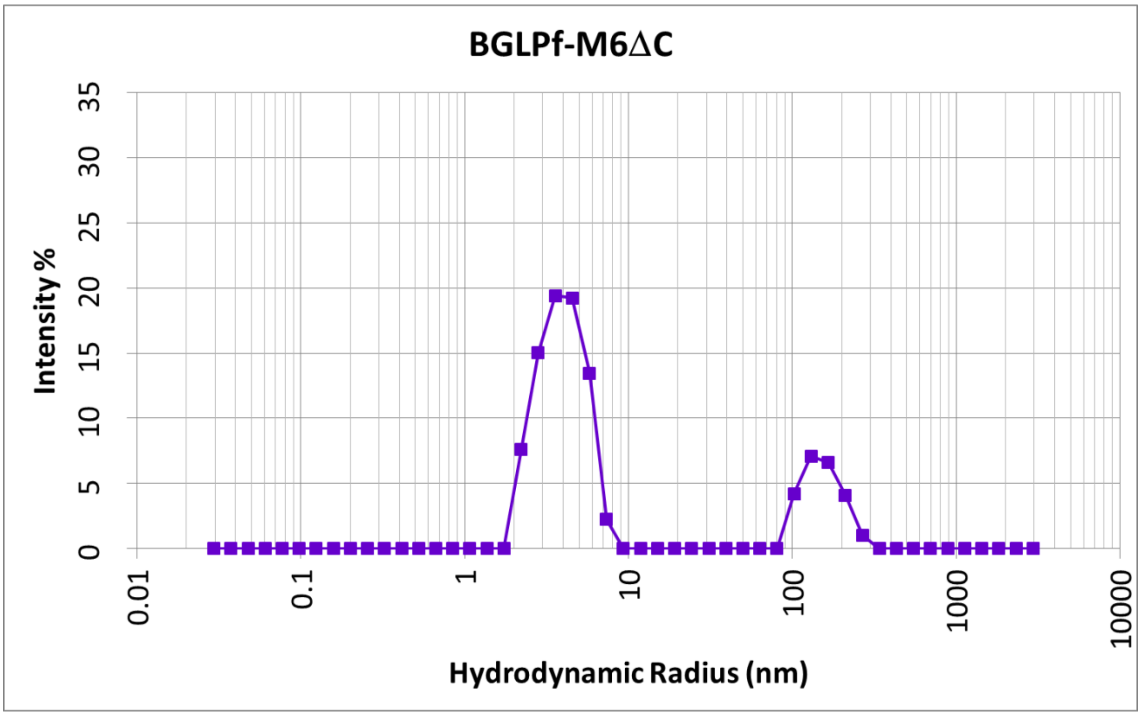

Figure S4 DLS size distributions of the BGLPf-M6ΔC. The result of the BGLPf-M6ΔC showed multimodal shaped distributions. The hydrodynamic radius is estimated to be 3.8 ± 1.2 nm.
